# Weaving together peer assessment, audios and medical vignettes in teaching medical terms

**DOI:** 10.5116/ijme.564a.2ed6

**Published:** 2015-12-06

**Authors:** Mohammad Allibaih, Lateef M. Khan

**Affiliations:** 1 Medical Terminology Unit, Faculty of Medicine, King Abdulaziz University, Saudi Arabia; 2 Department of Clinical Pharmacology, Faculty of Medicine, King Abdulaziz University, Saudi Arabia

**Keywords:** Medical terminology, medical history, medical errors, peer assessment, formative assessment

## Abstract

**Objectives:**

The current study aims at exploring the possibility of aligning peer assessment, audiovisuals, and medical case-report extracts (vignettes) in medical terminology teaching. In addition, the study wishes to highlight the effectiveness of audio materials and medical history vignettes in preventing medical students' comprehension, listening, writing, and pronunciation errors. The study also aims at reflecting the medical students' attitudes towards the teaching and learning process.

**Methods:**

The study involved 161 medical students who received an intensive medical terminology course through audio and medical history extracts. Peer assessment and formative assessment platforms were applied through fake quizzes in a pre- and post-test manner. An 18-item survey was distributed amongst students to investigate their attitudes and feedback towards the teaching and learning process. Quantitative and qualitative data were analysed using the SPSS software.

**Results:**

The students did better in the posttests than on the pretests for both the quizzes of audios and medical vignettes showing a t-test of -12.09 and -13.60 respectively. Moreover, out of the 133 students, 120 students (90.22%) responded to the survey questions. The students gave positive attitudes towards the application of audios and vignettes in the teaching and learning of medical terminology and towards the learning process.

**Conclusions:**

The current study revealed that the teaching and learning of medical terminology have more room for the application of advanced technologies, effective assessment platforms, and active learning strategies in higher education. It also highlights that students are capable of carrying more responsibilities of assessment, feedback, and e-learning.

## Introduction

Doctors are required to learn medical terminology, which is the language of communication between health care providers.^1^ Written, verbal, and electronic forms of communication depend totally on the medical language of which medical terminology is an essential component.

Working on preventing medical errors in the healthcare field remains justifiable considering the expensive and harmful endings they lead to, where for example 44000 - 98000 deaths and 1000000 excess injuries happen unnecessarily in the US each year.^2^^-^^4^

The application of powerful techniques and learning and teaching strategies in the medical education platform, especially the teaching and learning of medical terminology would certainly add a lot to the efforts being done to prevent or reduce the medical errors. Those errors are the ones emerging from the scriptural, typographical, and pronunciational mistakes the medical staff usually fall into.Although many researchers refer medical errors to various reasons, but spelling mistakes are still one of the obvious reasons a medical error could be made because of, starting from the spelling of the patient's identity.^5^^,^^6^Moreover, the part of bad medical term pronunciation makes it clear that miscommunication between health care professionals may result in medical errors. The use of the most advanced computer software in the field of computerized teaching and learning skills will lead to good practicing of medical terms, well-built background of practice, and a milestone of self-learning for medical students.^7^

The present pilot study aims at exploring the possibilities of integrating the audio facility in QuizCreator software, in the teaching of medical terminology to the second-year medical students. It also aims at reflecting the effect of applying the pair work strategy in a classroom of a large number of students to teach spelling and pronunciation skills from authenticated sources of medical terminology. It was obvious that our students lack the authenticated example of medical term pronunciation and writing, which if offered to them in such a challenging way, will in turn strengthen their reading, writing, and speaking skills.

This study aims to focus on the medical mistakes emerging from medical terminology spelling, and pronunciation mistakes done by health care providers such as physicians, nurses, technicians, etc. As whilst teaching using the paper-based technique in light of the pair-work strategy, almost unbelievable incompetence was demonstrated in the students' handling of the listening, speaking, and writing tasks. Freshmen are excused for looking confused and not able to adapt themselves with the new medical education field. Teller et al ^8^ relate that confusion to the medical field's busy, clamorous nature that is full of collisions of new conceptual frameworks, educational and pedagogical practices, strategies, and philosophies. There emerged the notion of providing students with tools that could extend their self-learning and e-learning outside classroom. Part of those tools was to offer virtual learning environments rich of valid and easy-to-access online contents. The thing that could come up with an enormous gain, enabling students to access the target knowledge whenever and wherever they may stand in need of.^9^

Furthermore, the study followed a collection of methods used to teach the medical terminology course to the students of the Faculty of Medicine at King Abdulaziz University in a way that could prevent or lessen their medical errors in the future. A close attention was focused on analogous medical terms that have a scriptural similarity and big difference in meaning expected to generate procedural, transcriptional, instructional, diagnosis and prognosis mistakes.

Moreover, learning medical terms is totally similar to studying a new language.^10^ A variety of teaching and learning strategies and techniques were applied to cure students' incompetence at the university level with a lot of training on two instructional forms; scriptural and pronunciational. The training covers the scriptural part through exposing students to handwriting with a special attention to spelling of paper-based as well as computerized instructions. True official instructional forms were used to target the adaptability on actual work proficiency.

### Objectives of the study

  1.    To develop medical terminology listening and writing skills of the medical students in their second year at the Faculty of Medicine, King Abdulaziz University.

  2.    To examine the impact of audio materials and actual medical history vignettes - given through an environment of active-learning strategies - on enhancing second-year medical students' listening and writing skills in medical terminology.

  3.    To participate in preventing or lessening the speaking and writing medical errors usually made by health care providers.

  4.    To widen the students' exposition to e-learning platforms through the features of the teaching and learning tools available in the technology used.

It is obvious that the learning outcome of the use of the aforementioned techniques (audios and medicinal extracts) is clearly set and well understood for students and institutions. Actually, this is aimed at meeting medical students' specific needs in medical terminology utilization for their specific healthcare field of study and future work requirements.

### Peer assessment concept

Peer assessment in higher education which is the practice whereby rating of peers is performed by a group of their colleagues, has been deployed for centuries although it appears as an innovative form of assessment.^11^^,^^12^ It was previously defined by Keith Topping as the arrangement or adaptation in which individuals (higher-education students) apply certain assessment criteria to judge their similar-status peers' learning outcomes or products.^13^ Topping summarizes that peer assessment appears as satisfactorily reliable and valid when utilized in various applications. Peer assessment is then proved for showing the positive formative impact on both attitudes and achievement of students to the extent that it is regarded as better than teacher assessment impact. Tim Swanwick focused some light on cautions when applying peer assessment for the reason that students are still on their way to be professionals and lack the solid knowledge to judge their peers' outputs.^14^

In Sadler's theoretical sense of formative assessment that is related to a broad spectrum of learning outcomes of a wide collection of subjects, students' output is judged with a simple correct-incorrect criteria.^15^ Sadler states that the theoretical definition of students' feedback is different in many significant respects with a particularly highlighted role in formative assessment from the definition usually found in the educational research. ^15^

Nevertheless, Epstein ^16^ argued that formative assessment adopts some assessment techniques whereas summative assessment adopts all techniques. The two forms of assessment contradict each other in the sense that formative assessment cares for the students' outcome of a program, while the summative assessment cares for students' assessment and the summary of their advancement. Both conceptual platforms of assessment are required by (future) physicians to identify their learning needs and respond to them.^16^

### Gains and benefits of peer assessment

Benefits and gains of peer assessment vary starting with providing feedback as an overriding target.^12^ Butler and Winne consider feedback as playing multiple roles in both teaching as well as learning processes including confirmation of pre-existing knowledge, acquisition of new knowledge, identification and correction of students' errors, improvement of information application, and helping wide restructure the schemata.^17^ Other possible benefits of peer assessment could be the great saving in instructor's time, improvement in the quality of work and subject being assessed, and enhancement in the quality of students' group work.^12^ additionally, practice tests used in formative assessment familiarize students with computer-managed learning and decrease their text anxiety.^7^Moreover, gains such as excellent classroom management, better utilization of devices and supplements, and achievement of the pre-set learning outcomes, although been regarded as great, will be considered as minor gains besides the aforementioned list of gains.

The teacher's role has been minimized in comparison with the students' role which has been maximized. Students in peer assessment almost do everything starting with getting prepared for quizzes, taking and retaking quizzes, correction of peers' work, following up on class discussion, playing the most important role of providing feedback. While the teacher's role tends to be limited to a facilitator who is responsible for preparation of study material, classroom management, analysis, and interpretation of results.

### Technology and tools of assessment

#### Authentic sources

Gardner and Miller defined authentic materials as those materials planned for purposes other than learning a language.^18^ They may be in various communication varieties. For instance, scriptural, audiovisual, or whatever forms not specifically intended for the development of language learning when created.^19^ Therefore, audios, videos, or medical extracts previously prepared for whatever purpose other than teaching and learning will count for enriching the learning process as an authentic source of medical terminology.

Authentic materials provide invaluable benefits to the learning process such as nourishing learning strategies and bringing about motivation and promotion for acquiring the new skills and competences. For instance, they are flexible to employ in various teaching situations and strategies.

National and international healthcare bodies are paying increasing attention to medical errors and consequently to patient safety. In that spirit, a patient-safety curriculum is provided by WHO to medical schools of which audiovisual tools and teaching summaries are included targeting the undergraduate level.^20^

Radley and Woottipong state that, at their initial stages of language learning students should learn listening.^2^^,^^19^ Moreover, the students' speaking skill improvement could also be subject to listening as a means of interaction, provided by the spoken language the students may be exposed to.

### Peer assessment based on grades, marks, or tests

Topping^13^ stated that his work on the review of peer assessment was the first; whereby he reviewed a collection of research models in which students (peers) award their colleagues grades or marks in a variety of complex outputs. Students considered the process as demanding, but reducing their anxiety. Similar subjects examples are writing and presentation projects.^21^^-^^23^

## Methods

### Study design

The current study has been carried out at the Faculty of Medicine, King Abdulaziz University, KSA, during semester 1 of the academic year 2014/2015. The course consisted of 40 hours of intensive teaching work in lecture, practical, and e-learning settings.

This study consists of assessment tests and a questionnaire involving students and no incentive was given to them for participation. Furthermore, the study did not involve patients, animals, or any data from them. It was carried out in accordance with the guidelines of 45 CFR 46.101(b) - Human Research Protection Program, hence exempted from obtaining institutional ethical approval. We have designed two sets of tests; an audio quiz about medical term pronunciation and a quiz about medical vignettes with questions about referring medical cases to their proper specialists. The first contained 50 short definition audios of around 10 to 20 seconds each along with five multiple-choice answers. The second contained 30 vignettes from daily medical life along with five multiple-choice answers. Both tests were designed in paper- and computer-based formats for each of which the students sat twice; once at the beginning of the medical terminology course, as a paper-based pre-test, and then in an online format as a computer-based post-test at the end of the training course.

The goal behind the pre-tests was to measure students' listening and comprehension skills, current level, and understanding of medical terms and usage. While the aim of the post-tests was to evaluate students' advancement in light of the use of active learning strategies and varied learning tools, and their impact on students' level and understanding of the given medical terms.

We have designed a questionnaire of 18 Likert-scale items aiming at reflecting the students' attitudes on the use of audios, medical history extracts (vignettes), and the teaching and learning process.

### Participants and sample size

Second-year medical students of the Faculty of Medicine at King Abdulaziz University are considered beginners in studying medical terminology. They pass the preparatory year as a pre-requisite to their basic medical sciences in the second year, part of which is the medical terminology course. Their medical terminology course aims at providing them with full knowledge related to their future field of education and workplace.

The study participants consisted of 161 medical students studying at semester one of the academic year 2014/2015 at the Faculty of Medicine, King Abdulaziz University, Jeddah, Saudi Arabia. Specifically, 161 medical students received medical terminology using the audio facility merged with a fifty-item quiz. While, 133 of the participants received medical terminology study cases using medical vignettes merged in a thirty-item quiz. Both quizzes were given in paper-based and computer-based formats. Both samples were selected from the participants in a simple random sampling manner.

A survey of 18 items with a 3-point-Likert-type scale was distributed amongst students to investigate their attitudes towards the learning process. The students who have been exposed to the training and underwent the quizzing treatment will of course form a group of 133 students (those who were exposed to the audio and vignettes quizzes) out of the whole group that consists of 161 students. Therefore, we got 120 responses to the questionnaire forming 90.22% of the minimal participants (133 students).

### Data collection

The sources of data obtained for this study were the students' scores in the pretests, posttests, and the questionnaire. The scores of the offline pretest and online posttest were collected, compared, and analyzed to check the students' advancement. The questionnaire was in an online setting so respondents' data were collected and analyzed to demonstrate their attitudes towards the teaching and learning process and the use of audios and medical vignettes in the teaching of medical terminology.

### Procedure and data analysis

The students were asked to take the paper-based pre-tests and exchange their papers with their colleagues for checking and correction of each other's mistakes. Peer review was performed while the test is being monitored on the data show and questions discussed with the whole class. At the end, students were asked to give marks and return the papers to their colleagues. The test papers with scores on them were collected and revised. In the tutorial session two weeks later, the scores were announced to students again aiming at bringing about motivation and enthusiasm for them to challenge their previous scores. Then students were asked to take the same tests as CBTs (computer-based test) or IBTs (internet-based test). We have analyzed the obtained data through the qualitative and quantitative statistical analysis using IBM SPSS statistics version 22. The t-test - using SPSS software - was utilized to check whether there is a statistical difference between the students' scores in the pretests and posttests.

The goal behind the questionnaire was to investigate the sample's perception and personal feedback on the use of audio and medical history (vignettes) extracts - as authentic learning materials - in the teaching of medical terminology. For this purpose, the questionnaire was managed to consist of 18 items with a three-point Likert's scale setting (satisfied = 3, neutral = 2, dissatisfied = 1). A pilot study was executed in 20 random students to check the validity of this questionnaire. Moreover, the data obtained from the questionnaire was then analyzed using the Cronbach's alpha coefficient in SPSS software as a measure of reliability and internal consistency of our survey scale items.

## Results

### Evaluation of the students' performance

Table 1 below shows a comparison of the mean scores the students got in the medical terminology pre-and post-tests with audio materials and medical history vignettes merged in them.

**Table 1:** Students' remarkable improvement on both quizzes: with audio extracts and medical vignettes, faculty of medicine, KAU, 2015, (N=161 and 133 respectively)

Test types		Mean	N	Std.Deviation	*t*	Sig. (2-tailed) *P Value*
Quiz with audio extracts	Pre-test	43.19	161	6.16	-12.09	.000
	Post-test	48.53	161	1.89		
Quiz with medical history extracts (Medical vignettes)	Pre-test	20.22	133	6.32	-13.60	.000
	Post-test	27.71	133	3.13		

### The audio quizzes

It was found that 43.19 was the students' average mean score of the pretest, and 48.53 was their average mean score for their posttest. The standard deviations were 6.16 and 1.89 for the pre- and post-tests respectively. The t-test result was -12.09.

It can be concluded that the students who took the audio quizzes did better in the post-test (m=48.53) than the pre-test (m=43.19) with a significance level of (p<0.05) leading to the conclusion that the students' level of medical terminology listening and pronunciation competencies improved after learning with the audios.

### The vignettes quizzes

A comparison of the students' mean scores in the medical terminology pre-and post-tests with the medical vignettes merged in them is demonstrated in (Table 1). It is obvious that 20.22 was the students' average mean score on the pre-test, whereas 27.71 was their average mean score in the post-test. The standard deviations for the pre-test and the post-test were 6.32 and 3.13 respectively, while the t-test result was -13.60.

The abovementioned statistical results revealed that the students who have been exposed to the medical terminology with the integration of the medical vignettes in their curriculum scored higher grades on the post-test (m = 48.53) than they did on the pre-test (m = 43.19) with a significance level of (p<0.05) concluding that their comprehension skill of medical terminology has significantly improved after the training. In fact the learning process was effective as students who attended the course did better on the posttests than the pretests as (p<0.05) on both tests. Moreover, the means of analysis revealed the students' advancement and opinions towards the methods of teaching used in the teaching and learning processes.

### Reliability test

The reliability statistics show that the Cronbach's alpha scale analysis was performed for 16 standardized items giving a coefficient .792, which means that more than 79% of the variability in a composite score by combining those 16 items, would be considered as true-score variances or internally consistent reliable variances.

**Figure 1:**
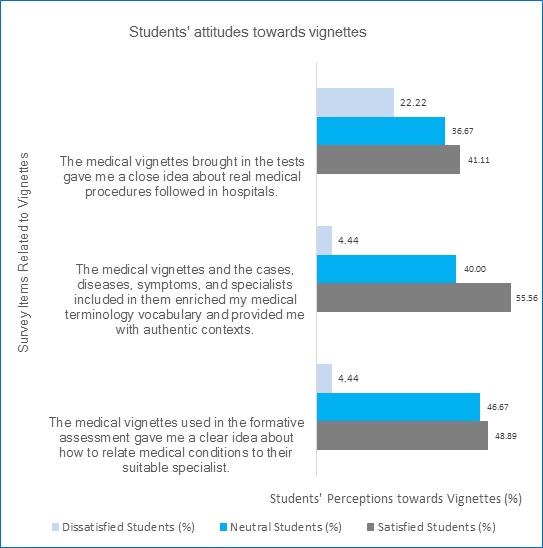
Students' perceptions towards using vignettes in the teaching and learning of medical terminology, faculty of medicine, KAU, 2015, (N=120)

Figure 1 demonstrates the students' perceptions towards the integration of vignettes in the teaching and learning of medical terminology.

It has been found that 55.56% of the students (n=67) considered that the medical vignettes and cases, diseases, symptoms, and specialists included in them have enriched their medical vocabulary and provided them with authentic contexts. Additionally, 48.89% of them (n=59) were satisfied with that the medical vignettes used in the formative assessment platform gave them a close idea of how to relate medical conditions to their appropriate specialists. In addition, medical vignettes were regarded by 41.11% of the students (n=49) as giving them a close idea about the actual medical procedures followed in hospitals.

**Figure 2:**
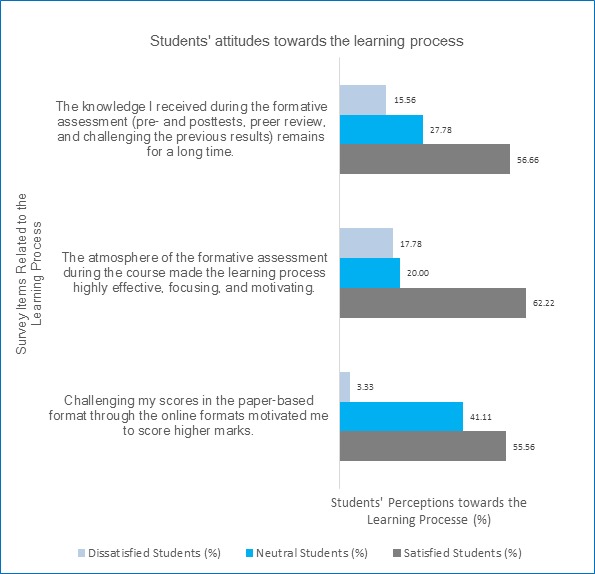
Students' perceptions towards the teaching and learning process, faculty of medicine, KAU, 2015, (N=120)

Students' attitudes towards the learning process and formative assessment are demonstrated in (Figure 2) below. It is obvious that 55.56% (n=67) to 62.22% (n=75) of the students are satisfied with the teaching and learning program. The survey reported that 56.66% of the students (n=68) are satisfied with that the knowledge they received remain for a long time by the aid of the formative assessment applied during the medical terminology course. In addition, 62.22% of the students (n=75) evaluate the learning atmosphere of the formative assessment as highly effective, focusing, and motivating. Nearly 56% percent of the students (n=67) agree with that challenging their scores in the paper-based quizzes by repeating the same quizzes online motivated them to score higher marks. The neutral opinions of students ranged between 20% (n=24) and 41.11% (n=49) gave neutral opinions towards the training.

**Figure 3:**
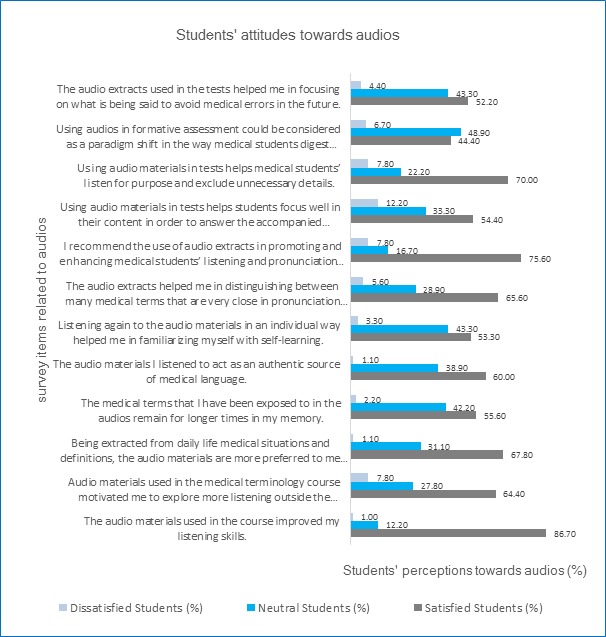
Students' perceptions towards using audio materials in the teaching and learning of medical terminology, faculty of medicine, KAU, 2015, (N=120)

Figure 3 shows the students' perceptions and attitudes towards the use of audio materials in the teaching and learning of medical terminology. It was found that 86.70% (n=104) of the students agree that the audios used in the course improved their listening skills. Moreover, 75.60% (n=91) of them recommended the use of audio materials in the teaching and learning of medical terminology as a tool to promote and improve listening and pronunciation skills of medical students. Additionally, 70% (n=84) of the students think that the audio materials helped them listen for purpose and exclude any unnecessary details. Many students 67.80% (n=81) prefer the audio materials extracted from daily life medical situations and definitions to the simple ones extracted from textbooks. It was also reported that 65.60% (n=79) of the students feel satisfied with that the audio extracts helped them distinguish between many medical terms with similarity in pronunciation and difference in meaning. On the other hand, 60% (n=72) of the students consider that the medical audios used in the training acted as an authentic source of medical language.

The study also revealed that 52.20% (n=63) of the students agree with that the audio extracts helped them focus on what is being said to avoid medical errors in the future. The survey results also reported that 55.60% (n=67) of the students are satisfied with that the medical terms they have been exposed to in the audios remain for a longer time. Additionally, 53.30% (n=64) of them agree with that listening again to the medical audios in an individual way helped them in familiarizing themselves with self-learning. The students 64.40% (n=77) agree that the audio materials motivated them to explore more listening outside the classroom. They 54.40% (n=65) also think that audios helped them focus well on their content in order to answer the accompanied questions correctly. Moreover, 44.40% (n=53) of them were satisfied with that the use of audio extracts in formative assessment looks like a paradigm shift in the way medical students digest knowledge.

## Discussion

The current study considers the student learning outcomes as performance criteria for curricula reformation in medical education and higher education as well. The study involved various teaching techniques including group discussions and large class activities depending on peer marking and peer review. The outcomes of this study meet further research outcomes based on methods such as group-oriented discussion, revealing that student-led discussions result in promising performance outcomes and fostered participation from students. That is beside student leadership ability and self-confidence.^24^

Consequently, the results drawn and the interpretations revealed from this study are partially in line with some other studies, such as the studies carried out by Wootitipong and McGuire.^19^ ^,^ ^25^ McGuire's study focused on the lexis of medical terminology and the strategies utilized to teach the corpus of medical terminology and how to decrypt and encrypt the different medical terms. On the contrary, Woottipong's study aimed at improving the listening skills of the students at the university level with the help of video materials.

Furthermore, another study conducted by Carpenter dealt with different teaching methods including lecture-discussion combination, team project, active learning strategies, lecture, and case study, in a large class setting. The study examined the effectiveness of the aforementioned teaching methods using inferential and descriptive statistical techniques in light of student learning outcomes.^26^Strikingly, the findings of the study revealed that the majority of the students most prefer lecture-discussion method. In our study, the students' positively reported their agreement to that the learning process as highly effective, focusing, and motivating by the application of formative assessment tools. Additionally, the score-challenging policy motivated the students to score higher marks.

It needs to be emphasized that, although the word authentic as a criterion is still moot, but the use of audiovisuals and medical vignettes in our study could be considered as an authentic material that is justified by the students' recommendation to be used in the teaching and learning of medical terminology at the university level. It is also worth mentioning that the students are satisfied with that the medical vignettes helped them having a clear idea on how to relate medical conditions to their suitable medical specialists, enriched their medical vocabulary with authentic contexts, and gave them a clear idea about the medical procedures followed in hospitals.

Indeed, the students are satisfied with that the audio materials are a paradigm shift in the teaching and learning of medical terms and a tool or a source of motivation, listening improvement, long-term memory refreshment, enhancement of listening and pronunciation skills, and help in excluding unnecessary details and distinguishing between the many terms that share close pronunciations.

### Limitations of the Study

This study has measured the medical students' ground standard of knowledge before the application of the assessment tools (pre- and post- tests) with the use of audio and medical history extracts (vignettes). It has also outlined the gained academic advancement the students got by the help of the aforementioned assessment tools in a formative assessment platform at the end of the medical terminology course. Moreover, the study has collected the students' perceptions regarding the whole teaching and learning process. One thing of an exceptional importance this study did not cover is the check of the long-term retention of the gained knowledge. Therefore, we recommend that future studies carry on an evaluation of long-term memory and long-term retention of the acquired knowledge.

### Implications of the study for medical education

It is of the utmost importance that medical education benefits a lot from the valuable feedback provided by formative assessment.^27^ Rushton stated that healthcare literature pays attention to feedback and formative assessment.^28^ The sort of frequent testing included in the formative assessment of this study was proved to enhance learning.^29^ Our study presents an experimental model of formative assessment (frequent testing) as well as positive students' feedback towards the use of medical audios medical vignettes in the teaching and learning of medical terminology. Similar courses in the healthcare and medical education fields could push forward the study with more comprehensive participation.

## Conclusions

Positive conclusions and interpretations can be obviously drawn from the aforementioned results obtained from the tests as well as the students' questionnaire during this study. It could be concluded that, using fake versatile quizzes in formative assessment along with tools such as active learning strategies, various testing formats, e-learning tools, and medical extracts and audiovisual aids, are effective in making medical terminology learning more engaging and efficient. This study has amply proved that the above-mentioned tools are more motivating, entertaining, and challenging than many other learning tools in higher education settings. It has been worthwhile that the atmosphere provided by the peer review and score-challenging concept is highly interactive and immersive for the fact that medical terminology is offered in an authentic context and through active learning strategies.

### Conflict of Interest

The authors declare that they have no conflict of interest.
